# Evaluating poor sleep quality and its risk factors in non-dialysis chronic kidney disease patients from Saudi Arabia

**DOI:** 10.1097/MD.0000000000043206

**Published:** 2025-07-11

**Authors:** Mohammed Alshehri, Weal Alshehri, Abdullah Alsubaie, Mohammed Asiri, Muath A. Alqahtani, Hassan Alasiri, Abdulsalam Asiri, Ali Alqahtani, Mushabbab Alqahtani, Abdullah Asiri

**Affiliations:** a Internal Medicine Department, College of Medicine, King Khalid University, Abha, Saudi Arabia; b Internal Medicine Department, Nephrology Section, Aseer Central Hospital, Abha, Saudi Arabia; c College of Medicine, King Khalid University, Abha, Saudi Arabia.

**Keywords:** chronic kidney disease, insomnia, Pittsburgh Sleep Quality Index, Saudi Arabia

## Abstract

This study aimed to assess sleep quality in patients with chronic kidney disease (CKD) in the Aseer Region of Saudi Arabia. This cross-sectional study focused on non-dialysis patients who attended CKD clinics. We used the validated Arabic version of the Pittsburgh Sleep Quality Index scoring questionnaire, which involves 7 component scores, each rated on a scale of 0 to 3. These component scores were aggregated to calculate a global score that ranged from 0 to 21. A global score >5 indicated poor sleep quality. The mean participants’ age was 53.8 ± 17.9 years, 66.5% were male, 93.5% were Saudi, 75.0% resided in high-altitude areas, 69.0% were married, 46% had an education level below the secondary level, 33.0% were employed, and 52.5% had a monthly income of <5000 Saudi Riyal. Regarding body mass index, 52.5% were overweight and 26% were obese. The global Pittsburgh Sleep Quality Index score among the study cases ranged from 1 to 17, with a mean score of 6.9 ± 3.3. In total, 123 (61.5%) were classified as poor sleepers and 77 (38.5%) as good sleepers. Predictors of poor sleep quality were age (adjusted odds ratio [AOR]: 1.40, *P* = .021), hypertension (AOR: 1.80, *P* = .046), diabetes mellitus (AOR: 1.64, *P* = .049), and estimated glomerular filtration rate (AOR: 0.78, *P* = .039). Most patients with CKD in our study experienced poor sleep quality. Factors such as age, history of comorbidities, and advanced CKD stages were associated with a higher likelihood of being a poor sleeper. Therefore, early screening and intervention for at-risk patients may be crucial for enhancing sleep quality among patients with CKD.

## 1. Introduction

Chronic kidney disease (CKD) is defined by the presence of kidney damage or a persistently reduced estimated glomerular filtration rate (eGFR) of <60 mL/min/1.73 m² for 3 months or longer.^[1]^ CKD leads to a gradual decline of kidney function, eventually leading to end-stage renal disease (ESRD).^[2]^ ESRD is characterized by an irreversible decline in kidney function, severe enough to be life-threatening without dialysis or transplantation.^[2,3]^

Sleep disturbances are a common complication in patients with CKD, especially in those with ESRD, affecting as many as 80% of patients.^[4]^ These disturbances, including insomnia, sleep apnea, restless leg syndrome, and excessive daytime somnolence, not only decrease quality of life,^[5,6]^ but are also linked to poor outcomes and an increased risk of mortality.^[4,7]^

Insomnia is a common condition characterized by difficulty in initiating or maintaining sleep and associated with poor sleep quality and quality of life quality,^[8]^ is notably more common in ESRD patients undergoing hemodialysis than in the general population, where it affects up to 30%.^[9]^ In patients with ESRD, the prevalence of insomnia symptoms ranges from 50% to 75%.^[10–13]^ This increased occurrence of sleep disorders among patients with ESRD can be attributed to several factors: a disruption of sympathovagal balance due to impaired baroreceptor reflex function, leading to increased sympathetic activity and reduced vagal tone^[14–16]^; lower levels of nocturnal melatonin^[13,14]^; and other factors related to kidney disease such as physical deconditioning, chronic inflammation, and pain.^[17]^ However, the full pathophysiological mechanisms underlying these sleep disturbances in patients with CKD are not completely understood.

The age-standardized prevalence of CKD (stages 1–2, stage 3, stage 4, and stage 5, excluding renal replacement therapy) in Saudi Arabia is estimated to be 9892 per 100,000, which is higher than the estimates for Western Europe (5446 per 100,000) and North America (7919 per 100,000).^[18]^ In Saudi Arabia, studies that focus on the relationship between kidney and sleep disorders have primarily centered on ESRD patients.^[12,19,20]^ This study aimed to fill the research gap by evaluating the prevalence of poor sleep among non-dialysis CKD patients and exploring the associations between various factors and poor sleep quality. Our findings could lead to more personalized interventions and improve our understanding of the CKD-sleep link, potentially influencing healthcare policies and practices in regions with similar CKD demographics.

## 2. Methods

This cross-sectional study was conducted in 2 centers the Aseer Region of Saudi Arabia from October 2022 to May 2023. The Aseer Region, located on a high plateau in southwestern Saudi Arabia, is an administrative area with Abha as its capital. Aseer is an administrative region in the southwestern part of Saudi Arabia, with Abha serving as its capital. Using G * power software, the minimum sample size required to assess the prevalence of sleep disorders among patients with CKD was 187. This assumption was made with a power of 80%, an alpha error of 5%, and a 59% prevalence of poor sleep quality among patients with CKD.^[13]^

Participants diagnosed with CKD, defined as having an eGFR of <60 mL/min/1.73 m² for 3 months or longer; adults aged 18 years or older; patients in various stages of CKD (stages 1–5), excluding those undergoing renal replacement therapy or post-kidney transplant patients; and the ability and willingness to provide informed consent to participate in the study.

We excluded patients with acute kidney injury or recent changes in kidney function that are not indicative of chronic disease; severe comorbid conditions, such as advanced cancer or severe psychiatric disorders, which could confound the results or severely impact sleep quality; pregnant women, due to potential changes in sleep patterns and CKD management during pregnancy; and individuals with significant cognitive impairment that could affect their ability to participate in surveys or sleep assessments.

We used a non-probability sampling technique, specifically a convenience sampling method, to gather participants for this study. Our study focused on individuals attending nephrology clinics who met the inclusion criteria.

We categorized patients with CKD based on the Kidney Disease Improving Global Outcomes CKD classification into 6 stages based on their eGFR. Stage G1 includes patients with an eGFR of 90 mL/min/1.73 m² or higher, with evidence of kidney disease, such as hematuria or proteinuria. Stage G2 corresponds to an eGFR between 60 and 89 mL/min/1.73 m², indicating mild CKD. Stage G3 is divided into G3a and G3b, with G3a representing an eGFR between 45 and 59 mL/min/1.73 m² (mild to moderate CKD) and G3b representing an eGFR between 30 and 44 mL/min/1.73 m² (moderate to severe CKD). Stage G4 includes patients with an eGFR between 15 and 29 mL/min/1.73 m², reflecting severe CKD, while Stage G5 includes those with an eGFR of <15 mL/min/1.73 m², indicating kidney failure.

## 3. Data collection

We used a predesigned structured questionnaire to collect patient data, which had 3 sections. The first section collected demographic information including age, sex, occupation, marital status, income in Saudi riyal (1 USD = 3.75 SAR), residency high altitude residency (higher than sea level) versus low altitude (lower than sea level). The second section collected medical information, such as the most recent serum creatinine level and medical history including diabetes mellitus (DM), hypertension (HTN), and heart disease. The most recent serum creatinine level was recorded for all patients. The Chronic Kidney Disease Epidemiology Collaboration equation was used to estimate the glomerular filtration rate (eGFR) of the participants.^[20]^


$$\begin{array}{l} \begin{array}{ll} & eGFR=142\times min\;(standardized\;Scr/K,1)\;\alpha \times max(standardized\;Scr/K,1)\;\\ & -1.200\times 0.9938\;age\;in\;years\times 1.012[if\;female]\; \end{array} \end{array}$$

where:

Scr = serum creatinine in mg/dL,

*K* = 0.7 (females) or 0.9 (males),

α = ‐0.241 (females) or ‐0.302 (males),

min (standardized Scr/*K*,1) = the minimum of Scr/*K* or 1,

max (standardized Scr/*K*,1) = the maximum of Scr/*K* or 1.

The second section contained a self-assessment tool comprising 19 questions focused on an individual’s sleep quality over the past month. The questions covered various aspects of sleep, including the time it takes to fall asleep, the duration of sleep, and the frequency and intensity of specific sleep-related issues. The 19 questions were grouped into 7 distinct components: subjective sleep quality, sleep latency, sleep duration, habitual sleep efficiency, sleep disturbances, use of sleeping medication, and daytime dysfunction. Each was assigned equal weight and scored on a scale of from 0 (no difficulty) to 3 (severe difficulty). The cumulative scores from these 7 components were then aggregated to calculate an overall Pittsburgh Sleep Quality Index (PSQI) score, which ranged from 0 to 21. A higher PSQI score is indicative of poor sleep quality. We categorized the total scores using a cutoff point of 5, where participants with a global score of 5 or less were considered good sleepers, indicating good sleep quality, while those with a score above 5 were categorized as poor sleepers, indicating poor sleep quality.^[21]^

In the third section, we calculated the body mass index (BMI). BMI was calculated using the formula:

BMI = weight (kg)/height (m)^2^, where weight was measured in kilograms and height in meters. The BMI categories were defined as follows: underweight (BMI < 18.5), normal weight (BMI 18.5–24.9), overweight (BMI 25.0–29.9), and obesity (BMI ≥ 30.0).

## 4. Data analysis

The data were collected and thoroughly reviewed, encoded, and input into International Business Machines Corporation Statistical Package for the Social Sciences statistical software, version 22 (SPSS, Inc., Chicago). All statistical analyses utilized two-tailed tests, with a *P*-value of <.05 considered statistically significant. We conducted a descriptive analysis that included the frequency and percentage distribution of demographics, medical history, and eGFR for all variables.

To analyze factors related to the sleep quality of the study participants, we used crosstabulation. The significance of the relationships in the cross-tabulation was assessed using Pearson chi-square test and exact probability test for smaller frequency distributions. Additionally, multiple stepwise logistic regression employing the backward likelihood ratio method was utilized to identify the most significant determinants of sleep quality. The adjusted odds ratios (AOR) and corresponding 95% confidence intervals were used to describe the association of dependent and independent variables.

## 5. Results

Two hundred participants completed the questionnaire. Participants’ ages ranged from 14 to 85 years, with a mean age of 53.8 ± 17.9 years, and approximately 38% were aged over 60 years. Most of the participants (66.5%, n = 133) were male, and (93.5%; 187) were Saudi. The dominant residence for most of the study participants was in high-altitude areas (75%, 150). A total of 138 (69%) participants were married and 38 (19%) were single. Regarding educational level, 92 (46%) had an education below the secondary level, 40 (20%) had completed high school, and 43 (21.5%) had a bachelor’s degree or higher. In terms of employment status, 66 (33%) were employed, 57 (28.5%) were unemployed, and 72 (36%) were retired. A monthly income of <5000 SAR was reported by 105 (52.5%) participants, while 30 (15%) had a monthly income exceeding 10,000 SAR. Regarding BMI, 105 (52.5%) were overweight, 52 (26%) were obese, and 43 (21.5%) had normal body weight (Table 1).

**Table 1:** Socio-demographic characteristics of study patients with CKD (n = 200).

Demographics	No	%
*Age in years*		
<40	47	23.5%
40–50	41	20.5%
51–60	36	18.0%
>60	76	38.0%
Mean ± SD	53.8 ± 17.9	
*Gender*		
Male	133	66.5%
Female	67	33.5%
*Nationality*		
Saudi	187	93.5%
Non-Saudi	13	6.5%
*Residence*		
High-altitude area	150	75.0%
Low-altitude area	50	25.0%
*Marital status*		
Married	138	69.0%
Single	38	19.0%
Divorced/widow	24	12.0%
*Educational level*		
No education	45	22.5%
Elementary school	47	23.5%
Secondary School	25	12.5%
High School	40	20.0%
Bachelor or higher	43	21.5%
*Occupation*		
Employed	57	28.5%
Unemployed	66	33.0%
Retired	72	36.0%
Student	5	2.5%
*Monthly income*		
<5000 SAR	105	52.5%
5000–10,000 SAR	65	32.5%
>10,000 SAR	30	15.0%
*Body mass index*		
Normal weight	43	21.5%
Overweight	105	52.5%
Obese	52	26.0%
Mean ± SD	27.9 ± 4.3	
*eGFR* (mean ± SD)	29.15 ± 17.39	

eGFR *=* estimated glomerular filtration rate, I USD = 3.75 SAR, SD *=* standard deviation.

The most common comorbidities were HTN (79%), DM (59%), heart disease (27%), anemia (26%), hyperlipidemia (24%), and chronic respiratory diseases (11%) (Table 3).

**Figure 1.**
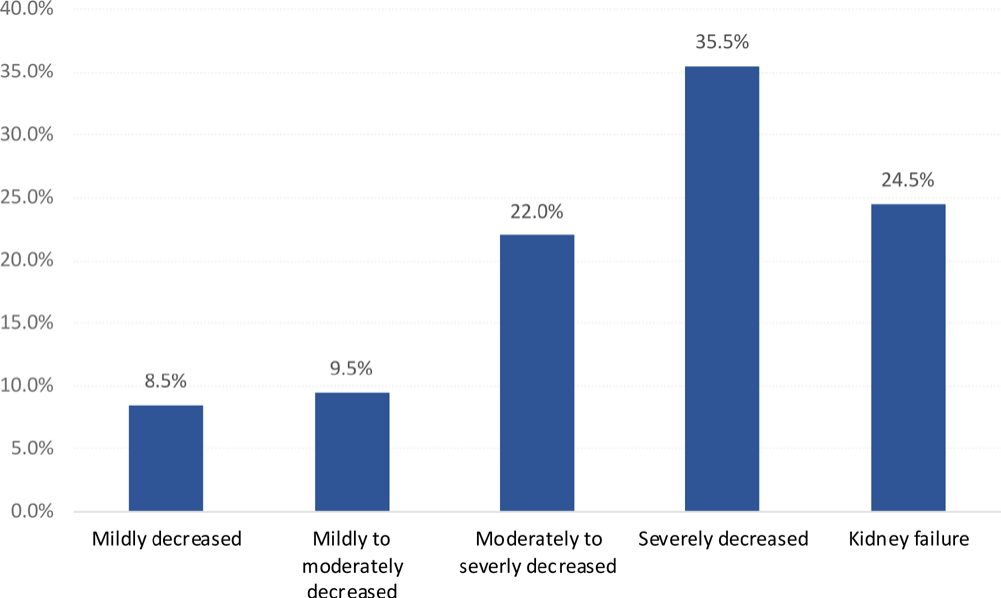
Kidney function based on reported eGFR among study patients with CKD. CKD = chronic kidney disease, eGFR = estimated glomerular filtration rate.

A total of 49 (24.5%) cases were classified as CKD stage 5, 71 (35.5%) had CKD stage 4, 44 (22%) had CKD stage 3b, and 19 (9.5%) had CKD stage 3a. Only 17 patients (8.5%) showed mildly impaired kidney function (Fig. 1).

The subjective sleep quality was poor in 21.0% of the study patients. Additionally, 19.5% experienced moderate to severe sleep latency and 23.5% had a sleep efficiency of <65%. Regarding sleep duration, 50% slept for <6 hours daily. Approximately 21.5% experienced moderate-to-severe sleep disturbance, 12.5% used sleep medication during the last month, and 33% experienced moderate-to-severe daytime dysfunction. The global PSQI score among the study cases ranged from 1 to 17 out of 21, with a mean score of 6.9 ± 3.3. A total of 123 (61.5%) participants were classified as poor sleepers and 77 (38.5%) as good sleepers (Table 2).

**Table 2:** Pittsburgh Sleep Quality Index components among study cases with CKD.

Sleep quality components	No	%
*Subjective sleep quality*		
Very good	62	31.0%
Fairly good	96	48.0%
Fairly bad	16	8.0%
Very bad	26	13.0%
*Sleep latency*		
None	78	39.0%
Mild	83	41.5%
Moderate	20	10.0%
Severe	19	9.5%
*Sleep duration*		
>7 hours	42	21.0%
6–7 hours	58	29.0%
5–6 hours	61	30.5%
<5 hours	39	19.5%
*Sleep efficiency*		
>85%	87	43.5%
75–84%	32	16.0%
65–74%	34	17.0%
<65%	47	23.5%
*Sleep disturbance*		
None	22	11.0%
Mild	135	67.5%
Moderate	39	19.5%
Severe	4	2.0%
*Use of sleep medication*		
Not during the past month	175	87.5%
Less than once a week	11	5.5%
Once or twice a week	9	4.5%
Three or more times a week	5	2.5%
*Daytime dysfunction*		
None	94	47.0%
Mild	40	20.0%
Moderate	48	24.0%
Severe	18	9.0%
*Global PSQI score*		
Range	1–17	
Mean ± SD	6.9 ± 3.3	

SD = standard deviation.

Table 3 shows that age, gender, nationality, residence, marital status, educational level, and occupation do not significantly affect sleep quality, as indicated by *P*-values above .05. However, monthly income, BMI, and co-morbidities exhibit significant associations with poor sleep quality. Specifically, a *P*-value of .006 indicates that monthly income is significantly associated with poor sleep quality, while a *P*-value of .048 shows significant differences in sleep quality among different BMI categories. Additionally, significant differences in sleep quality are noted with the presence of co-morbidities, particularly HTN and other conditions, with *P*-values of .014 and .049, respectively when compared to patients with no history of comorbid conditions. The findings suggest that economic status, weight, and health conditions are important factors, Table 3.

**Table 3:** Factors associated with CKD patients’ sleep quality.

Factors	Sleep quality				*P*-value
	Good sleepers		Poor sleepers		
	No	%	No	%	
*Age in years*					.249
<40	20	42.6%	27	57.4%	
40–50	13	31.7%	28	68.3%	
51–60	10	27.8%	26	72.2%	
>60	34	44.7%	42	55.3%	
*Gender*					.243
Male	55	41.4%	78	58.6%	
Female	22	32.8%	45	67.2%	
*Nationality*					.558
Saudi	71	38.0%	116	62.0%	
Non-Saudi	6	46.2%	7	53.8%	
*Residence*					.450
High-altitude area	60	40.0%	90	60.0%	
Low-altitude area	17	34.0%	33	66.0%	
*Marital status*					.105†
Married	59	42.8%	79	57.2%	
Single	13	34.2%	25	65.8%	
Widowed	5	20.8%	19	79.2%	
*Educational level*					.570†
No education	15	33.3%	30	66.7%	
Elementary school	19	40.4%	28	59.6%	
Secondary School	23	35.4%	42	64.6%	
Bachelor or higher	20	46.5%	23	53.5%	
*Occupation*					.592
Employed	23	40.4%	34	59.6%	
Unemployed	24	33.8%	47	66.2%	
Retired	30	41.7%	42	58.3%	
*Monthly income*					.006*
<5000 SAR	39	37.1%	66	62.9%	
5000–10,000 SAR	19	29.2%	46	70.8%	
>10,000 SAR	19	63.3%	11	36.7%	
*Body mass index*					.048*
Normal weight	14	32.6%	29	67.4%	
Overweight	47	44.8%	58	55.2%	
Obese	16	30.8%	36	69.2%	
*Co-morbidities*					
None	14	60.9%	9	39.1%	.014*
HTN	55	35.0%	102	65.0%	.048*
DM	40	33.9%	78	66.1%	.109
Others	39	33.1%	79	66.9%	.049*

DM = diabetes mellitus, P = Pearson *X*^2^ test.

† Exact probability test.

* *P* < .05 (significant).

In total, 65.3% of patients with stage 5 CKD were classified as poor sleepers, compared to 47.1% of those with stage 1 CKD (*P* = .048). Regarding individual PSQI components, only sleep duration was affected by CKD stages. Specifically, only 24.5% of those with stage 5 CKD slept for more than 7 hours daily, versus 42.1% of those with milder stages of CKD, Table 4.

**Table 4:** Association between CKD patients’ sleep quality and their kidney function based on eGFR.

Sleep quality	Kidney function (eGFR)										*P*-value
	Stage G2		Stage G3a		Stage G3b		Stage G4		Stage G5		
	No	%	No	%	No	%	No	%	No	%	
*Subjective sleep quality*											.194
Very good	9	52.9%	7	36.8%	13	29.5%	16	22.5%	17	34.7%	
Fairly good	4	23.5%	8	42.1%	25	56.8%	38	53.5%	21	42.9%	
Fairly bad	2	11.8%	1	5.3%	5	11.4%	6	8.5%	2	4.1%	
Very bad	2	11.8%	3	15.8%	1	2.3%	11	15.5%	9	18.4%	
*Sleep latency*											.139
None	8	47.1%	8	42.1%	12	27.3%	31	43.7%	19	38.8%	
Mild	4	23.5%	6	31.6%	27	61.4%	27	38.0%	19	38.8%	
Moderate	1	5.9%	3	15.8%	4	9.1%	5	7.0%	7	14.3%	
Severe	4	23.5%	2	10.5%	1	2.3%	8	11.3%	4	8.2%	
*Sleep duration*											.049*
>7 hours	3	17.6%	8	42.1%	9	20.5%	10	14.1%	12	24.5%	
6–7 hours	8	47.1%	2	10.5%	17	38.6%	17	23.9%	14	28.6%	
5–6 hours	5	29.4%	7	36.8%	9	20.5%	26	36.6%	14	28.6%	
<5 hours	1	5.9%	2	10.5%	9	20.5%	18	25.4%	9	18.4%	
*Sleep efficiency*											.346
>85%	10	58.8%	11	57.9%	19	43.2%	31	43.7%	16	32.7%	
75–84%	3	17.6%	3	15.8%	7	15.9%	11	15.5%	8	16.3%	
65–74%	0	0.0%	2	10.5%	7	15.9%	10	14.1%	15	30.6%	
<65%	4	23.5%	3	15.8%	11	25.0%	19	26.8%	10	20.4%	
*Sleep disturbance*											.829
None	1	5.9%	0	0.0%	5	11.4%	10	14.1%	6	12.2%	
Mild	13	76.5%	16	84.2%	31	70.5%	44	62.0%	31	63.3%	
Moderate	3	17.6%	3	15.8%	7	15.9%	16	22.5%	10	20.4%	
Severe	0	0.0%	0	0.0%	1	2.3%	1	1.4%	2	4.1%	
*Use of sleep medication*											.081
Not during the past month	15	88.2%	15	78.9%	39	88.6%	61	85.9%	45	91.8%	
Less than once a week	0	0.0%	2	10.5%	0	0.0%	7	9.9%	2	4.1%	
Once or twice a week	0	0.0%	2	10.5%	4	9.1%	2	2.8%	1	2.0%	
Three or more times a week	2	11.8%	0	0.0%	1	2.3%	1	1.4%	1	2.0%	
*Daytime dysfunction*											.240
None	14	82.4%	9	47.4%	21	47.7%	27	38.0%	23	46.9%	
Mild	1	5.9%	4	21.1%	10	22.7%	17	23.9%	8	16.3%	
Moderate	1	5.9%	3	15.8%	11	25.0%	21	29.6%	12	24.5%	
Severe	1	5.9%	3	15.8%	2	4.5%	6	8.5%	6	12.2%	
*Sleep quality*											.048*
Good sleepers	9	52.9%	9	47.4%	15	34.1%	27	38.0%	17	34.7%	
Poor sleepers	8	47.1%	10	52.6%	29	65.9%	44	62.0%	32	65.3%	

P = exact probability test.

* *P* < .05 (significant).

Age showed a significant positive association with poor sleep quality [AOR: 1.40, *P* = .021], indicates that for every 10-year increase in age, the odds of poor sleep increase by 40%. HTN is also significantly associated with poor sleep quality (AOR: 1.80, *P* = .046), suggesting that CKD patients with hypertension are more likely to experience poor sleep. DM had a borderline significant association (AOR: 1.64, *P* = .049), indicating a potential link with poor sleep quality. The eGFR is inversely associated with poor sleep quality (AOR: 0.78, *P* = .039), meaning better kidney function (higher eGFR) is associated with better sleep quality. Factors like marital status (unmarried) and other comorbid conditions showed trends toward significance, but their p-values are just above the traditional threshold (*P* < .05) (Table 5).

**Table 5:** Multiple stepwise logistic regression model for determinants of sleep quality among patients with CKD.

Determinants	*P*-value	AOR	95% CI	
			Lower	Upper
Age in years	*.021	1.40	1.01	5.80
Unmarried	.062	1.38	1.00	2.14
HTN	*.046	1.80	1.00	6.90
DM	*.049	1.64	.81	3.31
Others (CVD)	.062	1.92	.97	3.80
eGFR	*.039	0.78	0.41	0.97

AOR *=* adjusted odds ratio, CI *=* confidence interval, DM = diabetes mellitus.

* *P* < .05 (significant).

## 6. Discussion

Our study highlights significant sleep disturbances among pre-dialysis CKD patients. A majority of participants were identified as poor sleepers, with notable levels of severe sleep disturbances, use of sleep medication, and severe daytime dysfunction. Patients with advanced-stage CKD slept less than 7 hours per day, with their sleep duration affected by eGFR. Additionally, factors such as advanced age and comorbidities were found to contribute to poor sleep quality in this population.

Our study sheds light on sleep disturbances in patients with non-dialysis CKD. Approximately 61.5% of participants were poor sleepers, with an average global PSQI score of 6.9 ± 3.3. Approximately 21.5% reported severe sleep disturbances, 12.5% used sleep medication, and one-third suffered from severe daytime dysfunction. In the same vein, Yazici et al^[22]^ found that 42.5% of pre-dialysis CKD patients were poor sleepers, while our study indicated a higher prevalence, suggesting a potentially greater burden of sleep issues in our population. Furthermore, another cross-sectional study with 424 CKD patients included showed that 42.9% of screened patients had poor sleep quality.^[23]^ The disrupted sleep patterns observed in CKD patients may be attributable to the physiological changes caused by CKD. These might include alterations in melatonin metabolism or the accumulation of uremic toxins that affect sleep regulation. Additionally, psychosocial factors such as anxiety about disease progression or the burden of chronic illness could further exacerbate sleep disturbances. Physical symptoms such as pain, volume overload, and pruritus, which are more common in patients with CKD, may also contribute to poor sleep quality. These findings underscore the importance of addressing sleep quality in the management of patients with CKD. Implementing interventions such as cognitive behavioral therapy for insomnia or educating patients about healthy sleep habits should be considered. Moreover, identifying and addressing modifiable factors may offer pharmacological opportunities to enhance sleep quality in this population.^[24–26]^

Our study also identified various factors that contribute to poor sleep quality in patients with CKD, including advanced age, lower eGFR, comorbidities such as diabetes, hypertension, and obesity, as well as lower income levels. We observed notable parallels and distinctions when compared to the existing literature. A key finding of our study was the significant association between socioeconomic status and sleep quality, particularly with regard to income levels. This aligns with previous research, such as a cross-sectional survey of over 9000 participants, highlighting income-based sleep quality differences.^[27]^ Similarly, a study on low-income Chinese adults found that lower income and unemployment were significant contributors to poor sleep,^[28]^ mirroring our findings that 70% of lower-income patients were poor sleepers. Our study further contributes to the evidence linking comorbid conditions, specifically cardiovascular diseases, with sleep disturbance. This association resonates with the work of Lao et al,^[29]^ where a shorter sleep duration was significantly associated with a higher risk of coronary heart disease. Likewise, patients with newly diagnosed type 2 diabetes mellitus showed a strong link between sleep disturbance and an increased risk of cardiovascular diseases and all-cause mortality.^[30]^ These findings, although causality cannot be established, underscore the importance of managing sleep quality in patients with comorbid conditions, consistent with the observations of our study.

In addition to cardiovascular comorbidities, our study aligns with prior research that demonstrates a clear association between renal function deterioration and poor sleep quality.^[31,32]^ We observed significant differences in sleep quality across various grades of eGFR, with poorer sleep being more prevalent in patients with lower eGFR levels. This finding is consistent with several studies that have reported a correlation between declining kidney function and sleep disturbances, reinforcing the importance of monitoring sleep quality in patients with kidney impairment. The cause of CKD and the duration of the disease are important confounders that may affect sleep quality in CKD patients. For instance, patients with diabetic kidney disease are more prone to peripheral neuropathy and restless leg syndrome, both of which can alter sleep quality.^[33]^

Compared to pre-dialysis CKD patients, a study from Saudi Arabia on dialysis patients’ sleep quality reported that only 36% had poor sleep quality,^[19]^ which is lower than the results in our study. Another study,^[34]^ which included both pre-dialysis and hemodialysis patients, concluded that poor sleep quality was more prevalent in patients on dialysis, and age was not a strong predictor in either group. However, our study population was older than those in both studies, which may explain the difference in the prevalence and conclusions.

An important observation of our study relates to the effect of altitude on sleep quality. Aseer region, located in the south of Saudi Arabia, encompasses a wide range of altitudes, from sea level to 3000 m above sea level. Despite previous studies suggesting that high altitudes may affect sleep, our findings revealed no significant correlation between altitude and sleep quality. This contrasts with prior research indicating that altitude-induced changes in oxygen levels can impact sleep.^[35]^

The variation in predictors of poor sleep quality observed in our study underscores the need for individualized care in managing CKD patients. Given the impact of factors such as age, eGFR, comorbidities, and socioeconomic status, a standardized approach may not be sufficient. Clinicians should consider each patient’s unique clinical and socioeconomic circumstances when addressing sleep disturbances. Tailoring interventions (whether through patient education, behavioral therapy, or pharmacological treatment) can help improve sleep quality and potentially enhance overall patient outcomes.

## 7. Strengths and limitations

This study differs from previously published works in this region. It targets pre-dialysis CKD patients compared with ESRD patients in all previous studies. It also attempts to address the effect of living in high-altitude areas on sleep quality in patients with CKD. However, the cross-sectional design may introduce a recall bias. Another limitation of our study is the inability to determine the exact causes of CKD due to the cross-sectional study design. Additionally, we relied on patients’ self-reported data including living environments (high and low altitudes), which may not be entirely accurate. Specifically, cities on mountains were considered high altitude, while coastal or inland areas were considered low altitude. These limitations suggest the need for longitudinal studies and more precise environmental classifications to better understand the factors affecting sleep quality in CKD patients. Finally, convenience sampling is a common method used in studies because it is often the most practical choice when considering time, resources, and accessibility. However, this method can introduce some bias into the selection process, as participants are chosen based on their accessibility at clinics, rather than random or more complex probability-based methods.

## 8. Conclusions

The study highlights the significant prevalence of poor sleep quality among patients with CKD and the necessity of researching deeper into the association between kidney health, cardiac conditions, and socioeconomic factors that impact sleep patterns. Addressing sleep disturbances in this group of patients could potentially enhance their overall quality of life and health outcomes. Future research should focus on investigating the underlying mechanisms contributing to sleep issues in patients with kidney disease and exploring potential interventions to improve sleep quality.

## Acknowledgments

The authors extend their appreciation to the Deanship of Scientific Research at King Khalid University (KKU) for funding this work through a small-group project under grant number (RGP.1/197/44). The authors also acknowledge the assistance and guidance provided by the KKU Center for Medical and Health Research (CMHR).

## Author contributions

**Conceptualization:** Mohammed Alshehri, Weal Alshehri.

**Data curation:** Mohammed Alshehri, Weal Alshehri.

**Formal analysis:** Mohammed Alshehri.

**Investigation:** Mohammed Alshehri, Abdullah Alsubaie, Mohammed Asiri, Muath A. Alqahtani, Hassan Alasiri, Abdulsalam Asiri, Ali Alqahtani, Mushabbab Alqahtani.

**Methodology:** Mohammed Alshehri, Abdullah Alsubaie, Mohammed Asiri, Muath A. Alqahtani, Hassan Alasiri, Abdulsalam Asiri, Ali Alqahtani, Mushabbab Alqahtani.

**Resources:** Mohammed Alshehri.

**Software:** Mohammed Alshehri.

**Supervision:** Mohammed Alshehri, Abdullah Asiri.

**Writing – original draft:** Mohammed Alshehri, Weal Alshehri, Abdullah Alsubaie, Mohammed Asiri, Muath A. Alqahtani, Hassan Alasiri, Abdulsalam Asiri, Ali Alqahtani, Mushabbab Alqahtani, Abdullah Asiri.

**Writing – review & editing:** Mohammed Alshehri, Weal Alshehri, Abdullah Alsubaie, Mohammed Asiri, Muath A. Alqahtani, Hassan Alasiri, Abdulsalam Asiri, Ali Alqahtani, Mushabbab Alqahtani, Abdullah Asiri.
